# Mitochondrial microRNAs: Key Drivers in Unraveling Neurodegenerative Diseases

**DOI:** 10.3390/ijms26020626

**Published:** 2025-01-13

**Authors:** Raya Kh. Yashooa, Elisa Duranti, Donatella Conconi, Marialuisa Lavitrano, Suhad A. Mustafa, Chiara Villa

**Affiliations:** 1 Department of Biology, College of Education for Pure Science, University of Al-Hamdaniya, Mosul 41002, Iraq; raya.yashooa@uohamdaniya.edu.iq; 2 School of Medicine and Surgery, University of Milano-Bicocca, 20900 Monza, Italy; e.duranti@campus.unimib.it (E.D.); donatella.conconi@unimib.it (D.C.); marialuisa.lavitrano@unimib.it (M.L.); 3 General Directorate of Scientific Research Center, Salahaddin University-Erbil, Kurdistan Region, Erbil 44001, Iraq; suhad.mustafa@su.edu.krd

**Keywords:** microRNAs, mitochondria, neurodegenerative disorders

## Abstract

MicroRNAs (miRNAs) are a class of small non-coding RNAs (ncRNAs) crucial for regulating gene expression at the post-transcriptional level. Recent evidence has shown that miRNAs are also found in mitochondria, organelles that produce energy in the cell. These mitochondrial miRNAs, also known as mitomiRs, are essential for regulating mitochondrial function and metabolism. MitomiRs can originate from the nucleus, following traditional miRNA biogenesis pathways, or potentially from mitochondrial DNA, allowing them to directly affect gene expression and cellular energy dynamics within the mitochondrion. While miRNAs have been extensively investigated, the function and involvement of mitomiRs in the development of neurodegenerative disorders like Alzheimer’s disease, Parkinson’s disease, and amyotrophic lateral sclerosis remain to be elucidated. This review aims to discuss findings on the role of mitomiRs in such diseases and their potential as therapeutic targets, as well as to highlight future research directions.

## 1. Introduction

Neurodegenerative disorders (NDs) are a heterogeneous group of complex disorders characterized by neuronal cell death in the central or peripheral nervous system, resulting in movement, cognitive, and/or behavioral impairments [1]. The most common NDs include Alzheimer’s disease (AD), Parkinson’s disease (PD), and amyotrophic lateral sclerosis (ALS), each of which is associated with different genes but shares the characteristics of protein aggregate formation [2,3]. Accumulated evidence suggests that mitochondrial dysfunction contributes to the development of NDs, as neurons are particularly sensitive to mitochondrial homeostasis imbalances due to their high energy demand for neuronal growth, function, and regeneration [4,5]. Oxidative stress, excessive production of reactive oxygen species (ROS), and changes in calcium homeostasis in neurons can all result from an imbalance in mitochondrial homeostasis [6]. These factors can ultimately lead to neuronal apoptosis and play a role in the pathophysiology of NDs [7]. However, despite extensive research efforts in recent decades, the mechanisms underlying NDs remain unclear, and current treatments rely primarily on managing symptoms and halting disease progression [8].

Several studies have investigated the role of microRNAs (miRNAs) in mitochondrial dysfunction during the pathogenesis of NDs [9]. MiRNAs are a family of short, endogenous, non-coding RNA (ncRNA) molecules of approximately 22 nucleotides in length that negatively regulate gene expression either by targeting messenger RNA (mRNA) degradation or by suppressing protein translation [10]. MiRNAs are conserved regulators of various biological processes, and their aberrant expression or dysregulation has been associated with the pathogenesis of multiple diseases [11,12,13,14]. While the majority of mature miRNAs are typically located in the cytoplasm, some evidence has reported their presence in the mitochondrion, commonly referred to as mitochondrial miRNAs (mitomiRs), for their location of regulation [15,16]. Most of them are encoded by the nuclear genome and then translocate directly into the mitochondria, but some mitomiRs originate from mitochondrial DNA (mtDNA) [17]. They directly or indirectly modulate gene expression related to mitochondrial structure and function, specifically biogenesis, bioenergetics, and dynamics [18,19]. Since mitomiRs regulate key mitochondrial tasks, examining them could aid in the diagnosis, prognosis, and therapeutic targeting of NDs.

Hence, this review aims to explore the current knowledge of mitomiRs and their possible role in the pathophysiology of NDs, including AD, PD, and ALS. We further suggest future research directions to better characterize mitomiRs and their potential as therapeutic targets for NDs.

## 2. MitomiRs in Neurodegenerative Diseases

### 2.1. Biogenesis, Regulation, and Function of MitomiRs

**Figure 1:**
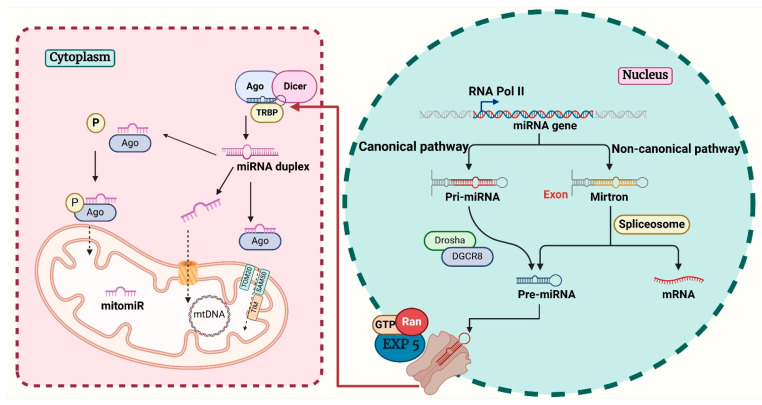
Schematic representation of mitomiR biogenesis. The majority of miRNAs are transcribed in the nucleus and then transported to the cytoplasm as pre-miRNAs. Further, mature miRNAs are generated upon cytoplasmic processing of pre-miRNA. Certain miRNAs are translocated to the mitochondria through different mechanisms. Additionally, some mitomiRs can originate from mtDNA. Created with BioRender.com. Ago, Argonaute; DGCR8, Di George critical region 8; Exp5, exportin 5; mitomiR, mitochondrial miRNA; mtDNA, mitochondrial DNA; pre-miRNA, precursor miRNA; pri-miRNA, primary miRNA; SAM50, sorting and assembly machinery 50; TIM, translocase of the inner membrane; TOM20, translocase of the outer membrane 20; TRBP, transactivation response RNA-binding protein.

MitomiRs may originate from the nucleus or be synthesized endogenously within the mitochondria, although the exact mechanisms of their biogenesis remain under investigation [15] (Figure 1). MiRNAs are frequently encoded either alone or in clusters within larger host genes. They may be found in introns, exons of protein-coding genes, or in the 3′-untranslated regions (3′-UTR). The remaining class of miRNAs is intergenic and transcribed separately from the host genes under their own promoters [20].

In the canonical pathway, miRNA is transcribed by RNA polymerase II/III into primary miRNA (pri-miRNA) within the nucleus [21]. It is then cleaved into hairpin structured precursor miRNA (pre-miRNA) by a nuclear microprocessor complex comprising an endonuclease RNA III enzyme named Drosha and an RNA-binding protein named DGCR8 (Di George critical region 8 or Pasha) that anchors Drosha to the pri-miRNA transcripts [22]. With its characteristic hairpin loop structure, pre-miRNA is highly stable and is then exported to the cytoplasm by exportin 5 (Exp5) and GTP-binding nuclear protein (RAN-GTP). Another RNAse III enzyme called Dicer processes the pre-miRNA into a mature double-stranded RNA (dsRNA) molecule, also known as the miRNA duplex [23]. Due to its greater thermodynamic stability, one of the single strands of the miRNA duplex is chosen to form the mature miRNA and binds to the RNA-induced silencing complex (RISC) composed of Argonaute (Ago1–4 in mammals) and related proteins in an ATP-dependent manner [24,25]. The negative regulation of gene expression is mediated by binding to the microRNA response element (MRE), which is primarily found in the 3′-UTR of mRNA, although it can also be located in the 5′-UTR or even in coding sequences [26,27]. Upon RISC binding to mRNA, the complex either promotes mRNA degradation or suppresses its translation, according to perfect or partial complementarity, respectively [28]. The ability of multiple miRNAs to target a single mRNA and a single miRNA to regulate multiple mRNAs reflects the complexity of miRNA-mediated regulation of key biological processes [29].

Due to their size and charge, mitomiRs cannot cross the double-membrane system of the mitochondria on their own; instead, they require molecular transport mechanisms. There are several hypotheses regarding how microRNA enters the mitochondria. One such postulation holds that Ago2 and the miRNA enter the mitochondria through the outer mitochondrial membrane via the pores SAM50 or TOM20 and then cross the inner mitochondrial membrane through TIM [30]. The import of miRNA into the mitochondria may also be mediated by other proteins, such as the exoribonuclease polyribonucleotide nucleotidyltransferase (PNPT1/PNPase) located in the intermembrane space of mitochondria, which regulates adenine nucleotide levels and mitochondrial homeostasis [31]. PNPase facilitates miRNA import by binding to the stem-loop motifs in the target RNA sequence. The diversity of imported miRNAs may be mediated by the regulation of amino acids within the KH and S1 domains of PNPase, which are known to have RNA-binding specificity [32,33]. It has also been hypothesized that Ago2 itself may act as a mitomiR import protein because of its capacity to bind RNA and its dual localization in the cytoplasm and mitochondria [30]. Additionally, the phosphorylation of Ago2 allows the Ago2-miRNA complex to enter cytoplasmic processing bodies (P-bodies), which interact with the mitochondria [30,34,35]. The voltage-dependent anion-selective channel protein (VDAC) may also play a role in miRNA import into mitochondria [36]. As VDAC is a highly conserved protein that is predominant in the outer membrane of mitochondria, it is thought to aid the translocation of small ncRNAs to the mitochondria [30,37,38]. Although plant mitochondrial VDAC has been demonstrated to bind tRNA and promote its import in vitro, its involvement in miRNA transport remains to be elucidated [39]. Apart from the canonical pathway, miRNAs/mitomiRs can originate from splicing introns (mirtrons) through the Drosha/DGCR8-independent non-canonical pathway. These pre-miRNAs are transported to the cytoplasm for Dicer processing and do not need to be processed by Drosha/DGCR8 [29]. Finally, mitomiRs can also be produced by the mitochondrial genome from its transcripts, although this hypothesis needs further investigation due to the absence of Dicer and Drosha enzymes [30]. The possible intramitochondrial biogenesis of miRNAs has been supported by the observation that sequences of certain pre-miRNAs and mature miRNAs exhibit alignment with the mitochondrial genome [15,40,41]. Similar to their nuclear and cytoplasmic counterparts, mitomiRs regulate gene expression by targeting the UTR of mitochondrial mRNAs [42]. A plethora of mitochondrial metabolic processes, such as the tricarboxylic acid (TCA) cycle, electron transport chain (ETC), oxidative stress, and nucleotide, amino acid, and lipid metabolism, have been linked to the function of mitomiRs. These pathways can be affected directly by influencing enzyme translation or indirectly by regulating the expression of transporters and regulatory proteins [43].

### 2.2. Dysregulated MitomiR in Neurodegenerative Disorders

#### 2.2.1. MitomiR in Alzheimer’s Disease

AD is the leading cause of dementia and is characterized pathologically by the intracellular formation of neurofibrillary tangles (NFTs) and extracellular accumulation of amyloid plaques (Aβ) in the brain [44,45,46]. These aberrant protein aggregates further promote inflammation, which exacerbates neuronal damage and accelerates the course of the disease [47,48]. Even though the exact pathophysiological process of AD is still unknown, mitochondrial dysfunction has been identified as an early and prominent hallmark of AD, which is consistent with the finding that impaired energy metabolism always occurs before disease onset [49,50]. Mitochondrial dysfunction in AD is characterized by a decline in ATP production, ROS overproduction, and influx of calcium ions, contributing to AD progression [51]. Specifically, reduced brain metabolism, particularly in the temporoparietal cortices of AD patients, has been shown to result in neuropsychological impairment and brain atrophy, as evidenced by neuroimaging analyses [52].

A study conducted by Kim and collaborators has reported high levels of miR-1273-g-3p in the cerebrospinal fluid (CSF) of early-stage-AD patients. This overexpression is associated with increased Aβ formation by inducing mitochondrial dysfunction and oxidative stress in AD model cell lines. The same authors also demonstrated that this miRNA negatively regulates the expression of the mitochondrial protein TIMM13 (translocase of inner mitochondrial membrane 13), thereby decreasing the activation of JNK (c-Jun N-terminal) and the expression of BACE1 (β-secretase 1), ultimately reducing Aβ generation. Additionally, TIMM13 was found to be downregulated in the brain postmortem of AD patients, suggesting its role as a potential marker for AD and a therapeutic target [53]. Another miRNA contributing to abnormal mitochondrial regulation is miR-195, which modulates the expression of mitofusin-2 (MFN2), a mitochondrial membrane protein that plays a central role in regulating mitochondrial fusion and cell metabolism. Using the senescence-accelerated mouse prone-8 (SAMP8) model of AD, the authors found increased levels of miR-195 along with decreased MFN2 in the hippocampus of mice, leading to disruption of the mitochondrial membrane potential [54]. This miRNA also targets and inhibits BACE1, stimulating Aβ production [55]. In a rat model of AD, it has been found that the overexpression of miR-140 downregulates the expression of PTEN-induced putative kinase 1 (PINK1), thereby enhancing mitochondrial dysfunction. Silencing of miR-140 resulted in decreased mitochondrial impairment and activated autophagy, as demonstrated by low levels of mTOR expression and tau phosphorylation, as well as increased levels of mitochondrial membrane potential, LC3-II/LC3-I, and Beclin 1 [56].

**Figure 2:**
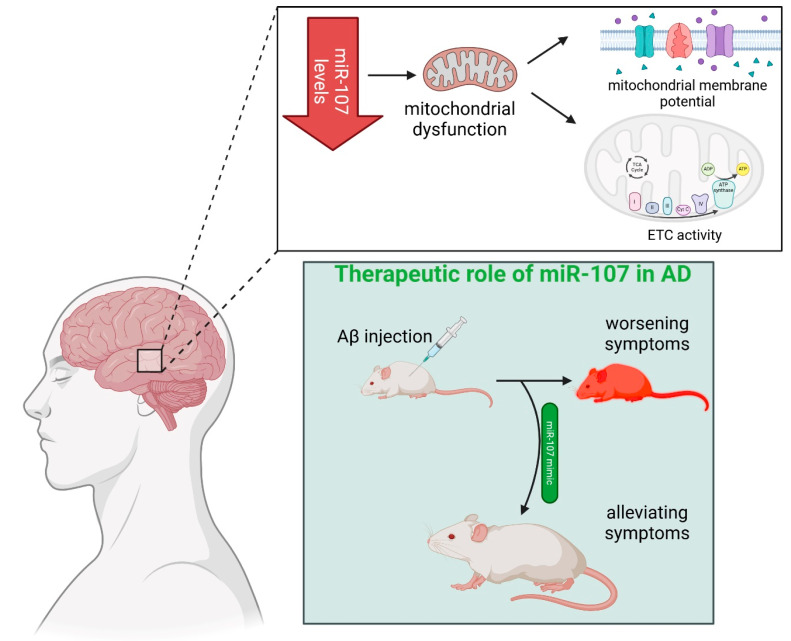
Recent evidence on the role of miR-107 in AD pathogenesis. Reduced miR-107 levels in the temporal cortex of patients with AD correlate with mitochondrial alterations. Suppressing miR-107 leads to a decline in mitochondrial membrane potential, whereas therapeutic restoration of miR-107 levels using a mimic reverses Aβ-induced AD-like symptoms in mouse models. Created with BioRender.com.

Another study reported that miR-107 levels were significantly decreased in the temporal cortex, even in patients with the earliest stages of AD [57,58]. Suppressing the expression of this miRNA led to a reduction in mitochondrial volume and cristae density, along with mitochondrial dysregulation, characterized by a decline in mitochondrial membrane potential and ETC activity (complexes I, III, IV, and V) [59]. A possible therapeutic role of miR-107 in AD was demonstrated in a study in which the acceleration of AD pathogenesis induced by Aβ injection, which resembles disease symptoms in mice, was reversed using a miR-107 mimic [60] (Figure 2). The activity of mitochondrial ETC complexes is enhanced by SIRT1, which deacetylates key transcription factors such as peroxisome proliferator-activated receptor γ coactivator-1α (PGC-1α), thereby improving cellular energy metabolism and reducing oxidative stress [61]. It has been reported that several miRNAs modulate *SIRT1* expression. In human brains of sporadic AD, upregulation of miR-9, miR-34a, miR-146a, and miR-155 was related to a decrease in SIRT1 [62,63]. Conversely, other studies have found low levels of miR-212/132 and miR-23a/23b clusters in the frontal cortex of patients with prodromal AD, resulting in overexpression of SIRT1 as a compensatory response. The increased activity is believed to enhance neuroprotective mechanisms against Aβ and tau pathology and oxidative stress [64].

Other miRNAs that may contribute to AD pathogenesis through mitochondrial dysfunction include miR-92a, miR-125b, miR-137, miR-143-3p, and miR-455-3p. A tau-induced transgenic mouse model of AD showed elevated levels of miR-92a, which targets the mitochondrial gene cytochrome b and disrupts γ-aminobutyric acid (GABA) transmission [65]. Similarly, in AD, upregulation of miR-125b, one of the most prevalent miRNAs in the brain, has been found [66,67]. This miRNA is closely associated with the regulation of tau phosphorylation, neuroinflammation, and cell death through modulation of oxidative stress and inflammatory factors, all of which are involved in the pathophysiology of AD [66,68,69]. The overexpression of miR-125b promotes tau phosphorylation by activating mitogen-activated protein kinase (MAPK), most likely by inhibiting its target phosphatase genes, *DUSP6* and *PPP1CA*. In C57BL/6 mice, injecting the miR-125b mimic directly into the hippocampus improved learning and memory, while inhibiting tau phosphorylation and expression of *DUSP6* and *PPP1CA* [69]. In addition, miR-125b also targets mitofusin 1 (MFN1), thereby altering mitochondrial dynamics [70]. Elevated levels of miR-143-3p were found in the serum from AD patients with AD, acting as a possible biomarker for the disorder [71]. Inhibiting this miRNA allowed neuronal protection in an in vitro AD cell model by targeting neuregulin-1 (NRG1), which plays an essential role in enhancing cellular oxidative capacity by controlling PGC-1α-mediated mitochondrial biogenesis [72,73]. Another miRNA involved in the regulation of mitochondrial dynamics is miR-137, which is downregulated in AD. Indeed, overexpression of this miRNA preserved primary cortical neurons against Aβ-induced neurotoxicity by targeting ERK1/2, suggesting a possible protective role [74]. Moreover, miR-137 regulated the expression of myocyte enhancer factor-2A (MEF2A), nuclear factor erythroid 2-related factor 2 (NRF2), and transcription factor A of mitochondria (TFAM) [75]. A similar protective role has also been reported for miR-455-3p against abnormal Aβ precursor protein (APP) processing and Aβ toxicity in a cellular model of AD. Additionally, treatment with this miRNA significantly reduced the levels of the fission genes *DRP1* and *FIS1* while increasing fusion proteins like OPA1, MFN1, and MFN2 [76].

#### 2.2.2. MitomiR in Parkinson’s Disease

PD is an ND characterized by resting tremors, slow movement, and muscle rigidity [77]. PD pathogenesis involves the degeneration of dopaminergic neurons in the substantia nigra pars compacta [78]. The disease is characterized by intracellular aggregation of α-synuclein (α-syn), shown as Lewy bodies and Lewy neurites [78,79,80]. PD is a complex disease, and its etiology is influenced by both environmental and hereditary factors. While the exact cause of PD remains unknown, age is considered the most significant risk factor [81]. The first association between mitochondrial dysfunction and PD was documented at the beginning of the 1980s, when the neurotoxin MPP+, a metabolite of MPTP (1-methyl 4-phenyl-1,2,3,6-tetrahydropyridine), induced Parkinsonism among patients by restricting NADH–ubiquinone oxidoreductase (complex I) of the mitochondrial respiratory chain [82,83,84]. Mitochondrial dysfunction in PD is considered an early marker of PD progression and a primary contributor to neuronal neurodegeneration [85]. The imbalance between ROS and mitochondrial function results in neuronal injury and oxidative stress [86]. ROS can damage macromolecules within neurons, including lipids, proteins, and nucleic acids, resulting in the degradation of dopaminergic neurons and alterations in neural networks [87]. Therefore, the relevance of mitomiRs in clinical settings is intriguing, notably their roles in oxidative stress, mitochondrial dysfunction [88], α-syn aggregation [89], neuroinflammation [90], and dysregulation of the intrinsic antioxidant system [91].

**Figure 3:**
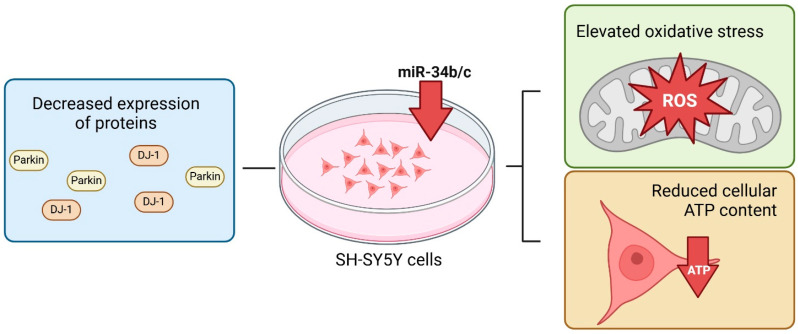
A summary of the influence of miR-34b/c depletion on mitochondrial function and PD development. Reduced miR-34b/c levels impair mitochondrial activity in differentiated SH-SY5Y cells, leading to increased oxidative stress and lower cellular ATP content. Downregulation of miR-34b/c is also associated with reduced expression of parkin and DJ-1, which are implicated in various forms of PD. Created with BioRender.com.

MiR-7 was reported to be downregulated in the brains of PD and MPTP-intoxicated animal models of PD [92,93]. It is documented that this miRNA protects neurons by inhibiting *SNCA*, the gene that encodes α-syn [92]. In response to MPP+-induced toxicity, miR-7 also inhibited the expression of VDAC1, consequently preserving neurons from mitochondrial fragmentation, depolarization, ROS production, cytochrome C release, and calcium efflux [94]. Furthermore, miR-7 suppressed the production of pro-apoptotic molecules and reduced neuron death in PD via targeting SIRT2 and Bax [95]. In addition to miR-7, also miR-34b/c regulates the expression of *SNCA* [96]. The depletion of miR-34b/c in differentiated SH-SY5Y dopaminergic neuronal cells led to altered function and dynamics of mitochondrial, elevated oxidative stress, and reduced cellular ATP content. Moreover, its downregulation was associated with decreased expression of parkin and DJ-1, two proteins linked to familial forms of PD, but also involved in idiopathic cases [96] (Figure 3). The mitochondrial direct target of miR-34b/c remains unknown, although a single nucleotide polymorphism (SNP) in the 3′-UTR of *SNCA* has been identified to diminish its repression via miR-34b/c [97]. The deglycase DJ-1 protein, predominantly located in the nucleus, cytoplasm, and mitochondria, prevents the aggregation of α-syn through its chaperone function under oxidative conditions [98]. miR-494 and miR-4639–5p target the 3′-UTR of DJ-1 and inhibit its levels in the brain [99,100]. Notably, miR-494 and miR-4639–5p levels were markedly elevated in the brains of patients with PD. The absence of DJ-1 correlated with diminished mitochondrial membrane potential, increased mitochondrial fragmentation, and the accumulation of autophagic markers in human dopaminergic cells [101,102]. In cellular and animal models of PD, miR-21 increased the expression of α-syn by targeting lysosome-associated membrane protein 2A (LAMP2A), which is necessary for the lysosomal clearance of α-syn aggregates [103].

In the MPTP model of PD, a previous study demonstrated low levels of miR-124, which is involved in the regulation of autophagy and apoptosis, two important cellular processes with complex and interconnected protein networks [104,105,106]. Interestingly, this miRNA was found to target Bim, which suppresses the translocation of Bax to the mitochondria and lysosomes, resulting in decreased apoptosis and impaired autophagy in dopaminergic neurons [105].

To preserve cellular homeostasis, damaged mitochondria must be removed by a complex mechanism called mitophagy, which is orchestrated by two post-translational modifications: ubiquitylation and phosphorylation [107]. In mammals, the most characterized mitophagy pathway is regulated by PINK1, a mitochondrial serine-threonine kinase that phosphorylates both ubiquitin and E3 ubiquitin ligase parkin (PRKN) [108]. The activation of PINK1 kinase and its mitochondrial localization sequence are essential for the translocation of parkin to depolarized mitochondria [109]. Conversely, PTEN dephosphorylates ubiquitin, blocking the activation of PINK1/PRKN-mediated autophagy [110]. MiR-27a and miR-27b have been shown to downregulate the expression of *PINK1* by targeting its 3′-UTR, preventing parkin from translocating to damaged mitochondria and inhibiting their degradation [111]. In a systemic rotenone model of PD, a previous study detected upregulation of miR-146a, which reduced the expression of parkin protein, thereby preventing the clearance of damaged mitochondria in neurons [112]. Another parkin target is miR-218, which negatively modulates PINK1/PRKN-mediated mitophagy [113]. This miRNA is also believed to inhibit NF-kB signaling by targeting *KPNA4* and is observed to be diminished in PD brains, ultimately leading to dysfunction of mitochondria [114].

Leucine-rich repeat kinase 2 (LRRK2) is another protein involved in mitochondrial function. Elevated LRRK2 protein levels can disrupt mitochondrial dynamics and integrity via dynamin-like protein (DLP1). Interestingly, the activity of miR-205 was significantly diminished in the brains of PD patients, concomitant with greater LRRK2 protein levels [115,116]. Subsequent research confirmed that miR-205 suppresses the expression of LRRK2 protein in primary neuron cultures and cell lines by targeting the 3′-UTR of the *LRRK2* gene [117].

Given the critical function of miRNAs in the development and progression of PD, short RNA molecules appear to be promising avenues for therapy, facilitating possible molecular targets, and enhancing individualized treatment strategies [118]. Valdés and Schneider developed small double-stranded RNA molecules that reproduce miRNAs and serve as gain-of-function tools for certain miRNAs [119]. These miRNAs can generate diminished target proteins by binding to the 3′-UTR of the mRNA of a specific target gene. Consequently, they can modulate genes and proteins linked to PD risk. Nonetheless, this technique may pose potential risks of off-target effects and the likelihood of negative interactions with other genes [119].

#### 2.2.3. MitomiR in Amyotrophic Lateral Sclerosis

ALS is a severe ND that affects motor neurons and nerve cells, which are crucial for controlling skeletal muscles and are essential for movement and life activities. As ALS progresses, it causes muscle weakness and atrophy, eventually impairing vital functions, such as breathing, leading to death [120,121]. The majority of ALS cases are sporadic, with no clear heritability (about 90–95%), while a small percentage are familial, often due to inherited genetic mutations. Among the different genes associated with ALS, the most well-known is the one encoding superoxide dismutase 1 (*SOD1*). Mutations in *SOD1* cause the accumulation of misfolded proteins, which damage nerve cells. In recent decades, thanks to advancements in molecular genetics, additional genes coding for transactive response (TAR)-DNA binding protein 43 (*TARDBP*), fused in sarcoma/translocated in liposarcoma (*FUS/TLS*), and chromosome 9 open reading frame 72 (*C9ORF72*) have been identified, suggesting a complex network of molecular alterations underlying ALS [3,122,123]. The pathogenesis of ALS is complex and includes multiple biological processes; however, mitochondrial dysfunction represents one of the major critical factors. Mitochondria play a crucial role in ATP production, intracellular calcium regulation, and management of ROS. In neurons and muscle cells, which demand high levels of ATP and stability to maintain their functions, mitochondria are essential for cellular health. When mitochondrial function is impaired, nerve cells become vulnerable to oxidative stress, which over time leads to degeneration and cell death [120,124,125].

**Figure 4:**
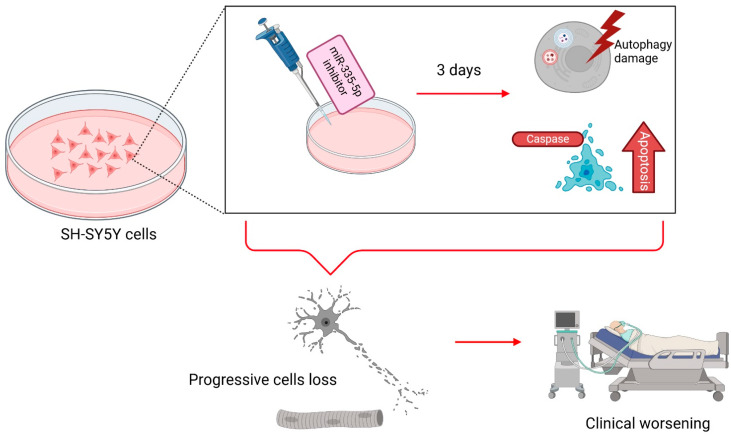
Role of miR-335-5p in ALS pathogenesis and mitochondrial regulation. MiR-335-5p dysregulation was observed in SH-SY5Y cells. Its depletion disrupts mitophagy, alters autophagy processes, and activates caspase 3/7-mediated apoptosis. These deficiencies contribute to the progressive loss of neuronal and muscle cell functioning, exacerbating ALS pathology. Created with BioRender.com.

Several studies have shown that specific mitomiRs are altered in patients with ALS, suggesting that these small ncRNAs may influence ALS pathology by disrupting mitochondrial function [126] (Figure 4). In intriguing studies, it has been shown that miR-335-5p, a mitomiR that regulates around 2500 genes, is significantly decreased in the serum of patients with ALS. Its reduction has been associated with impaired neuronal plasticity and damage to the process of mitophagy. In the same study, at 3 days post-transfection with an miR-335-5p inhibitor, SH-SY5Y cells displayed altered autophagy processes alongside the activation of caspase 3/7-mediated apoptosis pathways. This deficit may contribute to the progressive loss of functionality in nerve and muscle cells, worsening the condition of patients with ALS patients [126,127,128] (Figure 4). The link between impaired mitophagy and ALS was further supported by the observed upregulation of miR-27b-3p and miR-34a-5p in the CSF of patients with ALS and plasma samples of *C9ORF72*-carriers with ALS, respectively [129,130]. Both miRNAs suppress PINK1-mediated mitophagy by targeting the 3′-UTR of the *PINK1* gene [111,131].

It has been found that miR-129-5p is upregulated in cell and murine models of ALS harboring a *SOD1* mutation [132]. This miRNA downregulates the expression of the *ELAVL4* gene, which encodes the neuron-specific RNA-binding protein HuD critical for the regulation of several key neuronal mRNAs [133]. Interestingly, blocking miR-129-5p with an antisense oligonucleotide (ASO) resulted in improved motor function and increased survival in ALS mice [132]. Similarly, high levels of this miRNA have also been observed in the peripheral blood mononuclear cells (PBMCs) of sporadic ALS (sALS) patients, highlighting its potential as a therapeutic target for ALS [128,132,134]. In blood leukocytes, serum, CSF, and spinal cord samples from sALS patients, another study observed an upregulation of miR-338-3p [135], a brain-specific miRNA that reduces the expression of cytochrome C oxidase subunit IV (COX IV), resulting in decreased oxidative phosphorylation in the axons of neurons [136]. Additionally, in situ hybridization staining confirmed its expression and localization in the gray matter of spinal cord tissue samples collected from sALS autopsied patients [135].

In the skeletal muscle of ALS patients, the increase in miR-23a was associated with decreased mRNA and protein levels of PGC-1α, a master regulator of mitochondrial dynamics, through the control of the expression of core genes, including nuclear respiratory factors (NRFs), estrogen-related receptors (ERRs), MFN2, and COX IV [137,138]. Similar findings were also found in the skeletal muscle of SOD1-G93A transgenic mice, further strengthening the involvement of miR-23a in the dysregulation of skeletal muscle mitochondria [137]. Conversely, a protective role of miR-9 has been suggested, as its levels were downregulated in the spinal cord of ALS patients [139]. The overexpression of this miRNA has been associated with reduced mitochondrial damage and oxidative stress through the negative regulation of glycogen synthase kinase 3 β (GSK-3β), a protein found to be elevated in the spinal cords of ALS patients [140,141].

The possible role of miRNAs as prognostic biomarkers has also been investigated in other studies [128,142]. For instance, elevated levels of circulating miR-181 in the plasma of patients with ALS have been linked to ALS progression [143]. This miRNA inhibited parkin-mediated mitophagy and sensitized neuroblastoma SH-SY5Y cells to mitochondrial uncoupler-induced apoptosis [144]. Additionally, miRNA-181 was found to target anti-apoptotic proteins of Bcl-2 family members, including Bcl-2 and Mcl-1. Reduced levels of this miRNA were associated with decreased cell death, diminished oxidative stress, and improved mitochondrial function in astrocytes [145]. In addition, levels of miR-15b were downregulated in peripheral blood samples of sALS patients [142]. This miRNA acts as a negative regulator of sirtuin 4 (SIRT4), preventing ROS production and loss of mitochondrial membrane potential while modulating the mRNA levels of nuclear-encoded mitochondrial genes [146]. Furthermore, levels of miR-130a-3p, miR-151b, and miR-221-3p, which are reduced in the blood from patients with sALS, could be used in combination to monitor disease progression [142]. High levels of miR-206 have been detected in the affected muscles of SOD1-G93A mice, as well as in the blood plasma of these animals and serum samples from patients with ALS [147]. Notably, its levels were altered during disease progression, with the highest expression observed in the most severely affected animals, suggesting this miRNA as a possible prognostic candidate [147,148,149]. It has been hypothesized that the overexpression of miR-206 is a stress-related response to the disease since ALS mice defective in miR-206 exhibit an accelerated course of the disorder and impaired mitochondrial function [150]. This miRNA may play a compensatory role by promoting the regeneration of neuromuscular synapses and delaying disease progression through translation suppression of histone deacetylase 4 (HDAC4) [151]. Another miRNA correlating with disease progression is miR-124, which is upregulated in the brains of ALS mice at late disease stages [152]. Its overexpression affects motor neuron morphology and mitochondrial axonal transport by negatively regulating vimentin filaments [153,154].

## 3. Limitations and Challenges for MitomiR-Based Therapeutic Strategies

The development of miRNA-based therapeutics using miRNA mimics or inhibitors for NDs faces numerous challenges, including delivery methods, administration routes, dosage, and off-target effects [155]. Despite extensive preclinical investigations in murine models, few miRNA candidates have advanced to clinical development [156].

Effective delivery systems are essential, as miRNAs cannot passively diffuse across lipid membranes and are biologically unstable. Optimal miRNA delivery vehicles should have a high loading capacity, strong stability, long circulation half-life, gradual degradation of the miRNA payloads, and low toxicity. Currently, successful methods of miRNA delivery include non-viral and viral vectors [157,158]. The blood-brain barrier (BBB) poses further challenges for targeting brain cells, making nanoparticles a promising platform [159]. Targeted delivery of miRNA mimics or inhibitors to specific tissues or cells is difficult due to a variety of factors, including suboptimal cellular uptake, degradation, and lack of tissue selectivity. Nanoparticles address these concerns by protecting miRNAs and improving their therapeutic efficacy. However, their structural complexity and multi-step manufacturing procedures hinder their large-scale production and clinical translation. Simplifying nanoparticle designs and optimizing parameters like size, charge, and surface properties are crucial for developing effective, biocompatible carriers [160]. Exosomes, with their natural lipid bilayer architecture, are increasingly being recognized as effective carriers for miRNA delivery to the brain [161]. The viral system, including adeno-associated virus (AAV)- and lentivirus-based systems, is one approach for achieving long-term expression [158]. The AAV delivery method can maintain the expression of exogenous transgenes without integration into host genomes, whereas the lentivirus-based system produces transgenes constitutively due to genomic integration. Nonetheless, arbitrary incorporation of lentiviruses into genomes may interfere with endogenous gene expression [158].

While miRNAs can be delivered stereotaxically to specific regions, invasive procedures are not always allowed within the CNS [162]. To address this issue, modifying cell- or tissue-specific promoters when using viral delivery systems for miRNA transport is a possible alternative [163]. Another promising approach is the use of targeted exosomes, which can deliver miRNAs to specific tissues by targeting peptides [161,164].

MiRNAs regulate several genes, raising concerns about off-target effects [165,166]. For instance, miRNAs may target partially accumulated regions of unrelated transcripts, potentially resulting in undesired gene downregulation. Furthermore, their influence on non-target pathways raises concerns: miRNA-based therapies may have a deleterious effect on certain normal cellular processes since these molecules interact with a number of targets that are not associated with the disease. As a result, miRNA treatments may have unexpected adverse effects due to multiple interactions with direct targets [166]. To address these issues, it is necessary to develop personalized cocktails of miRNAs that target multiple pathways, potentially enhancing therapeutic efficacy while minimizing toxicity. These tailored cocktails should be generated by comprehensive profiling of mitomiR signatures in patient-derived tissues, such as blood, CSF, or induced pluripotent stem cells (iPSCs) [167].

Addressing these challenges is critical for advancing miRNA-based therapeutics. With continuing improvements in delivery systems and preclinical evaluations, these therapies can pave the way for successful clinical applications.

## 4. Conclusions and Future Perspectives

MitomiRs are crucial for regulating mitochondrial function and cellular homeostasis, especially in the brain, which experiences elevated energy demand and oxidative stress. The dysregulation of mitomiRs has been associated with the development of numerous diseases, including NDs such as AD, PD, and ALS (listed in Table 1), implying possible mitochondrial dysfunction in their pathogenesis [168].

MitomiRs affect critical cellular processes that are frequently disrupted in neurodegenerative diseases by regulating mitochondrial biogenesis, oxidative stress responses, mitophagy, calcium regulation, and apoptosis [169]. Recent data indicate that mitomiRs may also function as indicators for disease diagnosis and progression, as well as possible treatment targets. Nonetheless, considerable challenges remain in completely clarifying the mechanisms of action and translating these insights into practical applications.

Future investigations should focus on elucidating the precise functions of mitomiRs in pathological conditions, establishing reliable detection methodologies, and developing targeted therapeutics to regulate their activity. These improvements offer the potential to improve our comprehension of mitochondrial dynamics in NDs and facilitate the development of innovative diagnostic and treatment approaches. MitomiRs should be investigated to better understand their function in mitochondrial alterations and disease mechanisms. Key areas of study include non-invasive diagnostics, therapeutic techniques utilizing mitomiR, and the application of a systems approach to elucidate the link between mitomiR and other systems biology and omics technologies. The integration of omics technologies, such as transcriptomics, proteomics, and metabolomics, provides an unprecedented opportunity to comprehensively study the interactions between mitomiRs and other molecular networks in NDs. Systems biology approaches can be employed to construct predictive models that simulate the effects of mitomiR modulation on cellular and mitochondrial pathways. These models, when combined with high-throughput omics data, enable the identification of key regulatory hubs and interaction points within mitochondrial and cellular systems. This comprehensive perspective also facilitates the discovery of novel therapeutic targets by elucidating the interconnected roles of mitomiRs, mitochondrial dynamics, and cellular metabolism. Moreover, integrating multi-omics data can shed light on the temporal and spatial changes induced by mitomiRs in disease contexts, providing insights into how they influence both acute and chronic pathological processes. Investigating the cross-talk between mitomiRs and the broader transcriptomic and proteomic landscapes may also reveal previously unknown pathways involved in mitochondrial dysfunction, neurodegeneration, and therapeutic resistance [170]. Moreover, enhancing the understanding of mitomiRs implicated in neuroinflammation and immune responses will be beneficial. These efforts aim to enhance the use of mitomiRs as biomarkers and treatments for neurological diseases.

## Figures and Tables

**Table 1:** A summary of the most dysregulated mitomiRs in AD, PD, and ALS.

MitomiRs	Expression	Disorders	Key Target(s)	References
miR-7	↓	PD	*SNCA*	[92]
	↓	PD	*VDAC1*	[94]
	↓	PD	*SIRT2*	[95]
	↓	PD	*BAX*	[95]
miR-9	↑	AD	*SIRT1*	[62]
	↓	ALS	*GSK-3β*	[139,141]
miR-15b	↓	ALS	*SIRT4*	[142,146]
miR-21	↑	PD	*LAMP2A*	[103]
miR-23a	↑	ALS	*PGC-1α*	[137]
miR-23a/23b	↓	AD	*SIRT1*	[64]
miR-27a/b	Unknown	PD	*PINK1*	[111]
miR-27b-3p	↑	ALS	*PINK1*	[111,129]
miR-34a	↑	AD	*SIRT1*	[62,63]
miR-34a-5p	↑	ALS	*PINK1*	[130,131]
miR-34b/c	↓	PD	*SNCA*	[97]
miR-92a	↑	AD	*cytochrome b*	[65]
miR-107	↓	AD	ETC complexes I, III, IV and V	[59]
miR-124	↓	PD	*BIM*	[105]
	↑	ALS	*VIM*	[153,154]
miR-125b	↑	AD	*DUSP6, PPP1CA*	[69]
	↑	AD	*MFN1*	[70]
miR-129-5p	↑	ALS	*ELAVL4*	[132]
miR-137	↓	AD	*MEF2A*, *NRF2*, *TFAM*	[75]
miR-140	↑	AD	*PINK1*	[56]
miR-143-3p	↑	AD	*NRG1*	[72]
miR-146a	↑	AD	*SIRT1*	[62,63]
	↑	PD	*PRKN*	[112]
miR-155	↑	AD	*SIRT1*	[62]
miR-181	↑	ALS	*PRKN*	[144]
	↑	ALS	*BCL2*, *MCL1*	[145]
miR-195	↑	AD	*MFN2*	[54]
	↑	AD	*BACE1*	[55]
miR-205	↓	PD	*LRRK2*	[117]
miR-206	↑	ALS	*HDAC4*	[147,151]
miR-212/132	↓	AD	*SIRT1*	[64]
miR-218	Unknown	PD	*PRKN*	[113]
	Unknown	PD	*KPNA4*	[114]
miR-335-5p	↓	ALS	*CASP7*	[126]
miR-338-3p	↑	ALS	*cytochrome C oxidase*	[135,136]
miR-455-3p	↓	AD	*DRP1*, *FIS1*	[76]
miR-494	↑	PD	*DJ-1*	[100]
miR-1273g-3p	↑	AD	*TIMM13*	[53]
miR-4639–5p	↑	PD	*DJ-1*	[99]
